# Pathogenic variants in plakophilin-2 gene (*PKP2*) are associated with better survival in arrhythmogenic right ventricular cardiomyopathy

**DOI:** 10.1007/s13353-021-00647-y

**Published:** 2021-06-30

**Authors:** Elżbieta K. Biernacka, Karolina Borowiec, Maria Franaszczyk, Małgorzata Szperl, Alessandra Rampazzo, Olgierd Woźniak, Marta Roszczynko, Witold Śmigielski, Anna Lutyńska, Piotr Hoffman

**Affiliations:** 1 Department of Congenital Heart Diseases, National Institute of Cardiology, Alpejska 42, 04-628 Warsaw, Poland; 2 Molecular Biology Laboratory, Department of Medical Biology, National Institute of Cardiology, Warsaw, Poland; 3 Department of Biology, University of Padua, Padua, Italy; 4 Department of Demography, University of Lodz, Lodz, Poland; 5 Department of Medical Biology, National Institute of Cardiology, Warsaw, Poland

**Keywords:** Arrhythmogenic right ventricular cardiomyopathy, Desmosomal genes, Plakophilin-2, *PKP2*

## Abstract

Arrhythmogenic right ventricular cardiomyopathy (ARVC) is mainly caused by mutations in genes encoding desmosomal proteins. Variants in plakophilin-2 gene (*PKP2*) are the most common cause of the disease, associated with conventional ARVC phenotype. The study aims to evaluate the prevalence of *PKP2* variants and examine genotype–phenotype correlation in Polish ARVC cohort. All 56 ARVC patients fulfilling the current criteria were screened for genetic variants in *PKP2* using denaturing high-performance liquid chromatography or next-generation sequencing. The clinical evaluation involved medical history, electrocardiogram, echocardiography, and follow-up. Ten variants (5 frameshift, 2 nonsense, 2 splicing, and 1 missense) in *PKP2* were found in 28 (50%) cases. All truncating variants are classified as pathogenic/likely pathogenic, while the missense variant is classified as variant of uncertain significance. Patients carrying a *PKP2* mutation were younger at diagnosis (*p* = 0.003), more often had negative T waves in V1–V3 (*p* = 0.01), had higher left ventricular ejection fraction (*p* = 0.04), and were less likely to present symptoms of heart failure (*p* = 0.01) and left ventricular damage progression (*p* = 0.04). Combined endpoint of death or heart transplant was more frequent in subgroup without *PKP2* mutation (*p* = 0.03). Pathogenic variants in *PKP2* are responsible for 50% of ARVC cases in the Polish population and are associated with a better prognosis. ARVC patients with *PKP2* mutation are less likely to present left ventricular involvement and heart failure symptoms. Combined endpoint of death or heart transplant was less frequent in this group.

## Introduction

Arrhythmogenic right ventricular cardiomyopathy (ARVC) is a heart muscle disease characterized by fibrofatty replacement of the myocardium leading to electrical instability and ventricular arrhythmias and increasing risk of sudden death. Its prevalence in general population is estimated at 1 in 1000 to 1 in 5000 (Basso et al. 2009) as a result of possible underdiagnosed cases with mild or no symptoms. The diagnosis of ARVC is based on International Task Force Criteria and, since 2010, major diagnostic criteria include the presence of a pathogenic mutation (Marcus et al. 2010). At least 50% of cases are familial and ARVC is currently considered a genetically determined cardiomyopathy, mainly caused by pathogenic variants in genes encoding desmosomal proteins (Basso et al. 2009; Marcus et al. 2010).

The disease usually has an autosomal dominant pattern with age-related, incomplete penetrance and variable expression, leading to an isolated cardiac phenotype (Hoorntje et al. 2017). Pathogenic variants in desmosomal genes are being identified in 33 to 63% of probands (Gandjbakhch et al. 2018). Desmosomes are membrane protein complexes specialized for cell-to-cell adhesion and maintenance of the structural integrity of the ventricular myocardium. Defects in the structure of desmosomes result in cardiac myocyte detachment and death, gap junction remodeling, and dysregulation of the Wnt–beta catenin pathway, leading to fibrofatty tissue substitution and electrical instability (Gandjbakhch et al. 2018).

However, the genetic cause of ARVC remains unknown for 40 to 50% of patients (Gandjbakhch et al. 2018). It is reported that among gene-elusive cases, only about one fifth has a positive family history (Groeneweg et al. 2015). A high percentage of athletes among gene-elusive, non-familial ARVC subjects suggests that high-intensity exercise can play an important role in the disease pathogenesis (Sawant et al. 2014).

Among desmosomal genes, pathogenic variants in plakophilin-2 gene (*PKP2*) appear to be the most common cause of the disease accounting for 36 to 92% of mutations identified in desmosomal genes (Gandjbakhch et al. 2018). They are associated with an isolated right ventricular involvement and a conventional ARVC phenotype (Xu et al. 2017).

Despite a huge amount of data from many studies, a genotype–phenotype correlation is still lacking and predictive value of genetic testing is sometimes undermined (Sheikh et al. 2018). In the present study, we have evaluated the prevalence of *PKP2* variants in an ARVC cohort of Polish patients and investigated the genotype–phenotype correlation.

## Methods

### Study population

The study cohort comprised 56 patients fulfilling the 2010 International Task Force Criteria (ITFC) for ARVC, who were diagnosed at our institution during the years 1983–2019. As the National Institute of Cardiology is the reference center for ARVC in Poland, the study group consisted of patients from all over the country. All subjects were Caucasian and 48 (86%) were male. Each individual was screened for pathogenic variants in *PKP2*. The study was approved by the institutional bioethics committee and informed consent was obtained from each patient.

### Genetic testing

DNA was extracted from the peripheral blood by phenol extraction or salting-out method. All probands were screened for genetic variants in *PKP2*, the most frequently affected gene in ARVC patients (https://www.omim.org/entry/602861). Mutation screening was conducted by denaturing high-performance liquid chromatography (DHPLC) (Padua, Italy, tests performed between 2002 and 2005, *n* = 38) and/or next-generation sequencing (NGS) (Warsaw, Poland, test performed between 2015 and 2016, *n* = 37) of amplicons designed specifically to cover all *PKP2* exons including exon–intron boundaries.

NGS was performed using 454 Genome Sequencer (GS) Junior platform (Roche Diagnostics, Basel, Switzerland) according to the Amplicon Library Preparation Method Manual. All libraries were sequenced with minimum depth of 20 reads. Data were analyzed with GS Amplicon Variant Analyzer (AVA) software (Roche). DHPLC was performed with the use of Wave Nucleic Acid Fragment Analysis System 3500 HT with DNASep HT cartridge technology (Transgenomic Inc, Omaha, NE, USA).

Each suspected DHPLC eluate and/or Junior amplicon found in probands were confirmed with Sanger sequencing using 3500xL Genetic Analyzer (Life Technologies, Carlsbad, CA, USA) and BigDye Terminator v3.1 Cycle Sequencing Kit (Life Technologies) following the manufacturer’s instructions. Chromatograms were analyzed using Variant Reporter 1.1 (Life Technologies).

Variant frequencies were derived from gnomAD (http://gnomad.broadinstitute.org). VarSome database (https://varsome.com) was used for the bioinformatic prediction scores and evaluating variant’s classification according to the American College of Medical Genetics and Genomics (ACMG) criteria (Richards et al. 2015). The clinical significance of the variants was based on ClinVar (https://www.ncbi.nlm.nih.gov/clinvar). To evaluate genotype–phenotype correlation, we considered *PKP2* variants classified as pathogenic and likely pathogenic according to ACMG criteria.

### Clinical evaluation

The evaluation of patients involved detailed medical history, including age at diagnosis, family history, history of sports activity, arrhythmia (cardiac arrest, syncope, sustained and nonsustained ventricular tachycardia, premature ventricular beats, atrial fibrillation/flutter, appropriate implantable cardioverter-defibrillator (ICD) interventions, ventricular tachycardia ablation), and heart failure symptoms. ARVC diagnosis was based on the presence of major and minor criteria according to 2010 ITFC. A standard 12-lead electrocardiogram (ECG) was analyzed for negative T waves in precordial leads, QRS duration and dispersion, and presence of epsilon wave and notched S in leads V1–V3. Right ventricular dimensions, and right and left ventricular systolic function were obtained from two-dimensional echocardiographic examinations performed for clinical purposes. Patients were followed for clinical course (right and left ventricular damage progression) and combined endpoint (death or heart transplant).

### Statistical analysis

Clinical characteristics of patients were compared between patients with and without identified pathogenic variant in *PKP2*. For phenotype analysis, three patients with mutations in desmocollin-2, desmoglein-2, and desmoplakin genes (*DSC2*, *DSG2*, and *DSP*, respectively) were classified as *PKP2* mutation*-*negative. Continuous variables were expressed as means with SDs or medians with quartile deviation (QR) depending on symmetrical distribution or not, respectively. The normality of distribution was assessed using the Shapiro–Wilk test. Categorical variables were expressed as frequencies and percentages. Comparative analyses were done using the *χ*^2^ test or Fisher’s exact test for categorical data, as appropriate. Continuous variables were assessed with *t* test or Mann–Whitney *U* test for non-normally distributed variables. *P* values < 0.05 were considered statistically significant. Differences in survival probabilities were measured by Kaplan–Meier curve. The statistical significance of the mentioned differences was established by using the log-rank test. All statistical calculations were performed using the Statistica 12 package (Statsoft, Krakow, Poland).

## Results

### *PKP2* variants

**Fig. 1:**
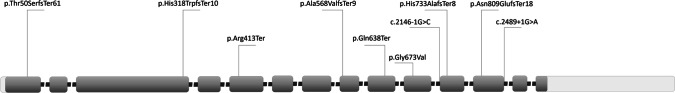
The distribution of *PKP2* variants found in this study

In 28/56 cases (50%, 89.3% male), variants in *PKP2* were identified. These included five frameshift, two nonsense, two splicing, and one missense variant; five of them were recurring variants (see Table 1 and Fig. 1). All truncating variants (frameshift, nonsense, splicing) are classified as pathogenic or likely pathogenic according to ACMG criteria. The only missense variant at the time, classified as variant of uncertain significance (VUS), has been taken into account on the basis of very low minor allele frequency in general population (gnomAD = 0.000) and the in silico prediction of pathogeneicity (predicted as deleterious by 10 prediction tools).

**Table 1:** *PKP2* (NM_004572.3) variants analyzed in our cohort

Variant (dbSNP ID)	Genomic coordinates (GRCh38)	ACMG verdict	ClinVar clinical significance	gnomAD allele frequency	Number of cases	Described
Frameshift variants						
p.Thr50SerfsTer61 c.148_151delACAG (rs397516997)	chr12-32,896,581-CTGT-	Pathogenic	Pathogenic	0	1	Yes
p.His318TrpfsTer10 c.929_951dupTGGATTCCAGCGGGAGGAGAGCG (rs1064792927)	chr12-32,877,929–CG…CA (23 bp)	Pathogenic	Pathogenic	0.00000407	2	No
p.Ala568ValfsTer9 c.1703delC (n/a)	chr12-32,824,148-G-	Likely pathogenic	n/a	0	1	No
p.His733AlafsTer8 c.2197_2202delCACACCinsG (rs397517021)	chr12-32,802,500-GGTGTG-C	Pathogenic	Pathogenic	0	1	Yes
p.Asn809GlufsTer18 c.2423dupA (n/a)	chr12-32,796,175–T	Likely Pathogenic	n/a	0	1	Yes
Nonsense variants						
p.Arg413Ter c.1237C > T (rs372827156)	chr12-32,850,907-G-A	Pathogenic	Pathogenic	0.00001415	4	Yes
p.Gln638Ter c.1912C > T (rs397517012)	chr12-32,822,526-G-A	Pathogenic	Pathogenic	0.000007074	3	Yes
Splicing variants						
c.2146-1G > C (rs193922674)	chr12-32,802,557-C-G	Pathogenic	Pathogenic	0.00003184	5	Yes
c.2489 + 1G > A (rs111517471)	chr12-32,796,108-C-T	Pathogenic	Pathogenic	0.00002829	9	Yes
Missense variants						
p.Gly673Val c.2018G > T (rs1426480515)	chr12-32,821,483-C-A	Uncertain Significance	n/a	0	1	Yes

### Genotype–phenotype correlation

**Fig. 2:**
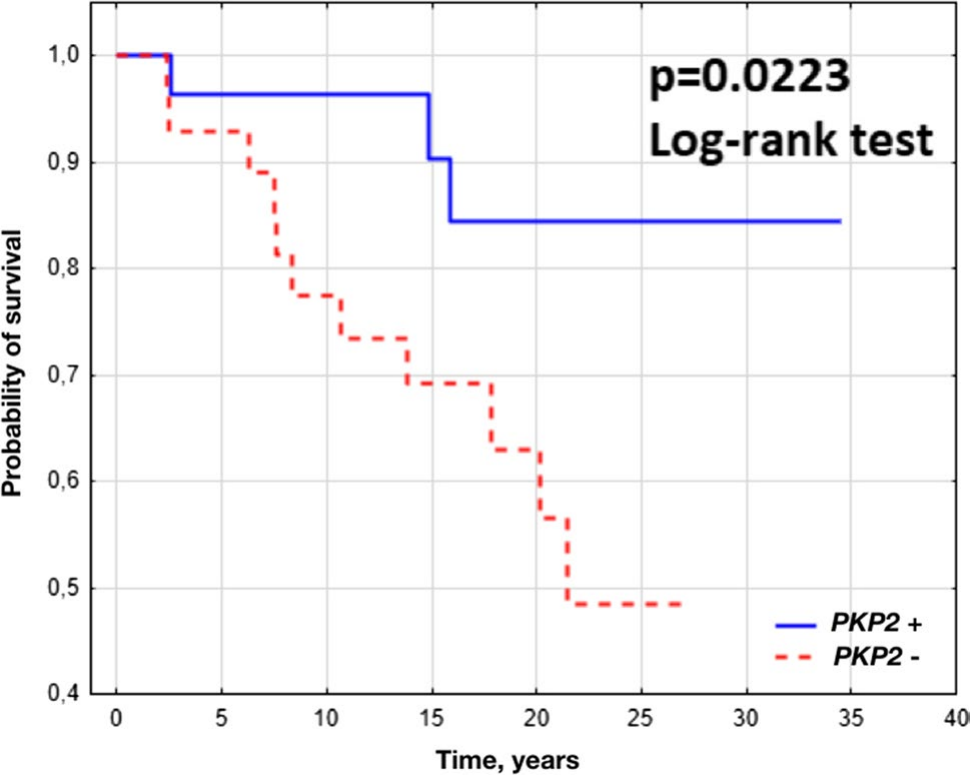
Kaplan–Meier survival curves for combined endpoint of death or heart transplant in *PKP2* mutation-positive and *PKP2* mutation-negative arrhythmogenic right ventricular cardiomyopathy patients

Table 2 summarizes the medical history, clinical features, arrhythmia, clinical course, and survival outcomes of *PKP2* mutation-positive patients compared with *PKP2* mutation-negative individuals. In *PKP2* mutation-positive group, all 28 patients were unrelated, while in *PKP2* mutation-negative group there were 27 probands and one third-degree relative. There was no difference between *PKP2* mutation-positive and mutation-negative individuals in sex, family history, history of sports activity, and arrhythmia (meaning cardiac arrest, syncope, ventricular tachycardia (VT), premature ventricular beats, supraventricular arrhythmias, ICD implantation, appropriate ICD interventions, and history of VT ablation) (see Table 2). At diagnosis, *PKP2* mutation-positive patients were significantly younger than *PKP2* mutation*-*negative subjects (mean ±SD age 32 ±11 vs. 41 ±12, *p* = 0.003). Total ITFC 2010 punctation was identical in both groups; however, negative T waves in leads V1–V3 occurred more often in *PKP2* mutation-positive patients (75% vs. 43%, *p* = 0.01), while epsilon wave was more frequent in *PKP2* mutation-negative subgroup (75% vs. 21%, *p* < 0.001). In echocardiography, right ventricular outflow tract dimension and presence of regional right ventricular akinesia or dyskinesia did not differ between those with and without identified *PKP2* mutations. Left ventricular ejection fraction (LVEF) was significantly lower in *PKP2* mutation*-*negative subjects (mean ±SD left ventricular ejection fraction 48 ±17 vs. 60 ±7, *p* = 0.04), which is consistent with the observation that individuals without a *PKP2* mutation were more likely to present symptoms of heart failure (54% vs. 21%, *p* = 0.01). Patients with and without identified *PKP2* mutations had similar proportions of right ventricular damage progression (68% vs. 68%; *p* = 0.83), while left ventricular damage progression was more often observed in *PKP2* mutation*-*negative patients (18% vs. 43%, *p* = 0.04). Combined endpoint of death or heart transplant occurred more frequently in *PKP2* mutation-negative group (11% vs. 39%, *p* = 0.03). This observation was confirmed by Kaplan–Meier survival analysis (see Fig. 2). However, when analyzing the causes of deaths divided into sudden death and death due to heart failure (this subgroup included also patients who underwent heart transplant), no significant differences were observed.

**Table 2:** Clinical characteristics of *PKP2* mutation-positive and *PKP2* mutation-negative arrhythmogenic right ventricular cardiomyopathy patients

	*PKP2* mutation-positive *n* = 28	*PKP2* mutation-negative *n* = 28	*p*
Male, *n* (%)	25 (89)	23 (82)	0.45
Age at diagnosis, years, mean (SD)	32 (11)	41 (12)	0.003
SCD in the young in the family, *n* (%)	4 (14)	4 (14)	1.0
ARVC in the family, *n* (%)	8 (29)	6 (21)	0.54
History of sports activity, *n* (%)	10 (36)	10 (36)	0.92
Cardiac arrest, *n* (%)	4 (14)	5 (18)	0.72
Syncope, *n* (%)	14 (50)	14 (50)	1.0
Sustained VT, *n* (%)	22 (79)	20 (71)	0.54
Nonsustained VT, *n* (%)	25 (89)	24 (86)	0.69
PVB > 500/day, *n* (%)	25 (89)	27 (96)	0.30
AF/AFL, *n* (%)	8 (29)	7 (25)	0.76
ICD, *n* (%)	20 (71)	15 (54)	0.17
Appropriate ICD interventions, *n* (%)	16 (57)	10 (36)	0.37
VT ablation, *n* (%)	14 (50)	15 (54)	0.79
Total ITFC punctation, mean (SD)	6.5 (1.5)	6.5 (1.7)	0.90
Negative T waves in leads V1–V3 (in the absence of RBBB), *n* (%)	21 (75)	12 (43)	0.01
QRS > 110 ms, *n* (%)	14 (50)	17 (61)	0.42
QRS dispersion, ms, mean (SD)	22 (22)	31 (19)	0.09
Epsilon wave, *n* (%)	6 (21)	21 (75)	< 0.001
Notched S in leads V1–V3, *n* (%)	15 (54)	13 (46)	0.31
RVOT PLAX, mm, mean (SD)	42 (8)	45 (12)	0.33
Regional RV akinesia or dyskinesia, *n* (%)	25 (89)	27 (96)	0.30
LVEF, %, mean (SD)	60 (7)	48 (20)	0.04
Heart failure symptoms, *n* (%)	6 (21)	15 (54)	0.01
RV damage progression, *n* (%)	19 (68)	19 (68)	0.83
LV damage progression, *n* (%)	5 (18)	12 (43)	0.04
Death or HTx, *n* (%)	3 (11)	11 (39)	0.03
Sudden death, *n* (%)	0 (0)	3 (11)	0.24
Death of heart failure or HTx, *n* (%)	3 (11)	8 (29)	0.18
Follow-up duration, years, mean (SD)	17.4 (9.1)	14.8 (8.0)	0.24

*AF* atrial fibrillation, *AFL* atrial flutter, *HTx* heart transplant, *ICD* implantable cardioverter-defibrillator, *ITFC* International Task Force Criteria, *LV* left ventricle, *LVEF* left ventricular ejection fraction, *PLAX* parasternal long-axis view, *PVB* premature ventricular beats, *RBBB* right bundle branch block, *RV* right ventricle, *RVOT* right ventricular outflow tract, *VT* ventricular tachycardia

## Discussion

ARVC is mainly caused by pathogenic variants in genes encoding desmosomal proteins, known as desmosomal genes. Among them, mutations in *PKP2* are the most common cause of the disease. In our study, *PKP2* mutations were identified in 50% of ARVC patients. This observation is consistent with previous studies estimating the frequency of *PKP2* mutations for 20–46% (James et al. 2020). Other desmosomal mutations are identified in 3–20% for *DSP*, 3–20% for *DSG2*, and 1–15% for *DSC2* (James et al. 2020).

### *PKP2* variants

The *PKP2* encodes plakophilin-2, a protein found primarily in cells of the myocardium. This gene belongs to the family of plakophilins characterized by numerous armadillo repeats, localizes to cell desmosomes and nuclei, and participates in linking cadherins to intermediate filaments in the cytoskeleton (Bass-Zubek et al. 2008; Cerrone et al. 2017).

In our cohort, nearly 97% of *PKP2* variants were truncating variants which is consistent with other data: according to ClinVar and VarSome databases, truncating variants account for 91.6 and 87.3%, respectively, of pathogenic/likely pathogenic variants in *PKP2*. The frequency of *PKP2* variants found in our cohort is consistent with their frequency in the gnomAD database, with two splice (c.2489 + 1G > A, c.2146-1G > C) and two nonsense (p.Arg413Ter, p.Gln638Ter) variants being the most common. However, one nonsense variant (p.Arg79Ter), which is also very often reported in the literature, was not found in our study group (Tintelen et al. 2006; Lint et al. 2019).

### Genotype–phenotype correlation

Despite many studies, the genotype–phenotype relationship in ARVC is not fully understood. It is believed that *PKP2* mutations are more likely to cause isolated right ventricular involvement and a conventional ARVC phenotype compared with other desmosomal mutations (Gandjbakhch et al. 2018; Riele et al. 2013). This thesis has been confirmed by our observations regarding the Polish group of ARVC patients.

As previously reported (Xu et al. 2017; Dalal et al. 2006), our results showed a younger age of onset in patients with *PKP2* mutation compared with those without *PKP2* mutation. As expected, we did not observe any differences between *PKP2* mutation-positive and negative individuals in the proportion of males. Although many previous studies indicated that the presence of desmosomal gene mutations as well as *PKP2* mutations is associated with a higher incidence of ventricular arrhythmias (Xu et al. 2017; Dalal et al. 2006; Ohno et al. 2013; Bao et al. 2013), this observation was not confirmed by our results. Also, Bhonsale et al. in a large USA/Dutch registry (Bhonsale et al. 2015) did not detect such correlation. However, it is worth noting that our results showed a higher occurrence of T-wave inversion in V1–V3 leads in *PKP2* mutation-positive patients than in *PKP2* mutation-negative subjects. This has also been demonstrated by the meta-analysis performed by Xu et al. in 2016 (Xu et al. 2017).

Many previous studies suggest that desmosomal mutations other than *PKP2* are more often associated with biventricular cardiomyopathy or isolated left ventricular involvement (Gandjbakhch et al. 2018; Bhonsale et al. 2015; Rigato et al. 2013; Castelletti et al. 2017; Fressart et al. 2010; Wong et al. 2019). Moreover, patients with these mutations, especially *DSG2* mutation carriers, present a higher risk of developing end-stage heart failure than *PKP2* mutation carriers (Gandjbakhch et al. 2018; Hermida et al. 2019). However, not all studies confirm this outcome (Bhonsale et al. 2015). Presence of *PKP2* mutation in our population was associated with higher left ventricular ejection fraction compared with mutation-negative individuals. Moreover, we found that ARVC patients with a *PKP2* mutation present less frequently left ventricular damage progression and symptoms of heart failure than subjects without *PKP2* mutation.

Unlike other researchers, we observed a higher incidence of epsilon wave in *PKP2* mutation*-*negative individuals compared with *PKP2* mutation carriers. A possible explanation of this discrepancy could be a more extensive myocardial injury in *PKP2* mutation*-*negative subgroup. It may also be related to the small size of the study group.

Observing our patients for years, we have an impression that *PKP2* mutation-positive subgroup presents more frequently classical ARVC phenotype, while *PKP2* mutation-negative subgroup includes patients showing clinical features ranging from benign ventricular arrhythmia to severe biventricular involvement leading to end-stage heart failure. Although subjects without *PKP2* mutation meet ITFC 2010, the diagnosis is less certain. Moreover, we found that in subjects without *PKP2* mutation combined endpoint of death or heart transplant occurred more frequently, which has not been clearly shown in previous studies.

### Study limitations

The study was conducted retrospectively; therefore, there was no consistent follow-up protocol. Genetic testing was performed in two laboratories at different times. Some patients were examined a few years ago and then they were lost to follow-up for various reasons. Due to the small size of the study group, we decided to include these patients. As a part of genetic testing, some patients were screened only for mutations in *PKP2*, while others also had other desmosomal genes tested. In three patients mutations in other ARVC-associated genes (*DSC2* /ACMG-VUS/, *DSG2* /ACMG-pathogenic/, and *DSP* /ACMG-VUS/) were found—these patients were classified as *PKP2* mutation negative. Furthermore, not all patients underwent myocardial biopsy.

It is worth noting that when analyzing the phenotype of ARVC patients, *PKP2* mutation-negative group consists of subjects with other desmosomal or non-desmosomal mutations, as well as patients without any mutation. The possibilities of comparing the phenotype of these subgroups are limited because they are too small for statistical analysis. In this context, it seems reasonable to compare patients with and without PKP2 mutation since the size of these groups is similar.

## Conclusions

Pathogenic variants in *PKP2* are responsible for 50% of ARVC cases in the Polish population. The presence of these variants is associated with a better prognosis and a lower incidence of combined endpoint, including death or heart transplant. ARVC patients with *PKP2* mutation are less likely to have left ventricular involvement and heart failure symptoms.

## Data Availability

All data and materials support the published claims and comply with field standards.

## References

[CR1] Bao J, Wang J, Yao Y (2013). Correlation of ventricular arrhythmias with genotype in arrhythmogenic right ventricular cardiomyopathy. Circ Cardiovasc Genet.

[CR2] Basso C, Corrado D, Marcus FI (2009). Arrhythmogenic right ventricular cardiomyopathy. Lancet.

[CR3] Bass-Zubek AE, Hobbs RP, Amargo EV (2008). Plakophilin 2: a critical scaffold for PKC alpha that regulates intercellular junction assembly. J Cell Biol.

[CR4] Bhonsale A, Groeneweg JA, James CA, et al (2015) Impact of genotype on clinical course in arrhythmogenic right ventricular dysplasia/cardiomyopathy-associated mutation carriers. Eur Heart J ehu509 10.1093/eurheartj/ehu509 25616645

[CR5] Castelletti S, Vischer AS, Syrris P (2017). Desmoplakin missense and non-missense mutations in arrhythmogenic right ventricular cardiomyopathy: genotype–phenotype correlation. Int J Cardiol.

[CR6] Cerrone M, Montnach J, Lin X (2017). Plakophilin-2 is required for transcription of genes that control calcium cycling and cardiac rhythm. Nat Commun.

[CR7] Dalal D, Molin LH, Piccini J (2006). Clinical features of arrhythmogenic right ventricular dysplasia/cardiomyopathy associated with mutations in plakophilin-2. Circulation.

[CR8] Fressart V, Duthoit G, Donal E (2010). Desmosomal gene analysis in arrhythmogenic right ventricular dysplasia/cardiomyopathy: spectrum of mutations and clinical impact in practice. Europace.

[CR9] Gandjbakhch E, Redheuil A, Pousset F (2018). Clinical diagnosis, imaging, and genetics of arrhythmogenic right ventricular cardiomyopathy/dysplasia: JACC State-of-the-Art Review. J Am Coll Cardiol.

[CR10] Groeneweg JA, Bhonsale A, James CA (2015). Clinical presentation, long-term follow-up, and outcomes of 1001 arrhythmogenic right ventricular dysplasia/cardiomyopathy patients and family members. Circ Cardiovasc Genet.

[CR11] Hermida A, Fressart V, Hidden-Lucet F (2019). High risk of heart failure in desmoglein-2 mutation carriers in arrhythmogenic right ventricular dysplasia/cardiomyopathy. Europace.

[CR12] Hoorntje ET, Te Rijdt WP, James CA (2017). Arrhythmogenic cardiomyopathy: pathology, genetics, and concepts in pathogenesis. Cardiovasc Res.

[CR13] James CA, Syrris P, van Tintelen JP, Calkins H (2020). The role of genetics in cardiovascular disease: arrhythmogenic cardiomyopathy. Eur Heart J.

[CR14] Marcus FI, McKenna WJ, Sherrill D (2010). Diagnosis of arrhythmogenic right ventricular cardiomyopathy/dysplasia: proposed modification of the Task Force Criteria. Eur Heart J.

[CR15] Ohno S, Nagaoka I, Fukuyama M (2013). Age-dependent clinical and genetic characteristics in Japanese patients with arrhythmogenic right ventricular cardiomyopathy/dysplasia. Circ J.

[CR16] Richards S, Aziz N, Bale S (2015). Standards and guidelines for the interpretation of sequence variants: a joint consensus recommendation of the American College of Medical Genetics and Genomics and the Association for Molecular Pathology. Genet Med.

[CR17] Rigato I, Bauce B, Rampazzo A (2013). Compound and digenic heterozygosity predicts lifetime arrhythmic outcome and sudden cardiac death in desmosomal gene-related arrhythmogenic right ventricular cardiomyopathy. Circ Cardiovasc Genet.

[CR18] Sawant AC, Bhonsale A, Te Riele ASJM (2014). Exercise has a disproportionate role in the pathogenesis of arrhythmogenic right ventricular dysplasia/cardiomyopathy in patients without desmosomal mutations. J Am Heart Assoc.

[CR19] Sheikh N, Papadakis M, Wilson M (2018). Diagnostic yield of genetic testing in young athletes with T-wave inversion. Circulation.

[CR20] Te Riele AS, James CA, Philips B (2013). Mutation-positive arrhythmogenic right ventricular dysplasia/cardiomyopathy: the triangle of dysplasia displaced. J Cardiovasc Electrophysiol.

[CR21] van Lint FHM, Murray B, Tichnell C (2019). Arrhythmogenic right ventricular cardiomyopathy-associated desmosomal variants are rarely de novo. Circ Genom Precis Med.

[CR22] van Tintelen JP, Entius MM, Bhuiyan ZA (2006). Plakophilin-2 mutations are the major determinant of familial arrhythmogenic right ventricular dysplasia/cardiomyopathy. Circulation.

[CR23] Wong JA, Duff HJ, Yuen T (2014). Phenotypic analysis of arrhythmogenic cardiomyopathy in the Hutterite population: role of electrocardiogram in identifying high-risk desmocollin-2 carriers. J Am Heart Assoc.

[CR24] Xu Z, Zhu W, Wang C (2017). Genotype–phenotype relationship in patients with arrhythmogenic right ventricular cardiomyopathy caused by desmosomal gene mutations: a systematic review and meta-analysis. Sci Rep.

